# The role of social networks in the self-management support for young women recently diagnosed with breast cancer

**DOI:** 10.1371/journal.pone.0282183

**Published:** 2023-04-13

**Authors:** Ivaylo Vassilev, Sharon Xiaowen Lin, Lynn Calman, Josh Turner, Jane Frankland, David Wright, Claire Foster

**Affiliations:** 1 School of Health Sciences, University of Southampton, Southampton, United Kingdom; 2 ARC Wessex, University of Southampton, Southampton, United Kingdom; 3 Management School, Xian Polytechnic University, Xian, China; 4 Centre for Psychosocial Research in Cancer: CentRIC^+^, School of Health Sciences, University of Southampton, Southampton, United Kingdom; The Hong Kong Polytechnic University, HONG KONG

## Abstract

It is widely acknowledged that social network support plays an important role in the quality of life and illness management of breast cancer survivors. However, the factors and processes that enable and sustain such support are less well understood. This paper reports baseline findings from a prospective UK national cohort of 1,202 women with breast cancer (aged <50 years at diagnosis), recruited before starting treatment, conducted in 2016–2019. Descriptive, univariate and multivariate regression analyses explored associations between the individual, and network member characteristics, and the type of support provided. Social network members provided a substantial level of illness-related, practical and emotional support. Highest contribution was provided by friends, followed by close family members. The social network members of women who did not have a partner provided a higher level of support than those in networks with a partner. Women without higher education were more reliant on close family members than those with higher education, and this was more so for women without a partner. Women with higher education without a partner were more reliant on friends and were overall best supported. Women without higher education who did not have a partner were overall least well supported. They had much smaller networks, were highly reliant on close family members, and on high level contributions from all network members. There is a need to develop network-based interventions to support people with a cancer diagnosis, prioritising support for the groups identified as most at risk. Interventions that support engagement with existing network members during treatment, and those that help extend such networks after treatment, are likely to be of benefit. A network perspective can help to develop tailored support and interventions by recognising the interactions between network and individual level processes.

## Introduction

The proportion of people surviving cancer has increased in recent decades, meaning that cancer is now considered a long-term or chronic condition [1]. This has impacted on treatment and follow up care pathways, which have shifted to include emphasis on self-management across the cancer care continuum [1]. In the United Kingdom, there has been a strategic shift to the delivery of personalised cancer care that is responsive to patient-identified need from the point of diagnosis, including support for self-management [2–4].

The role of social networks in the self-management of long-term conditions (LTCs) is well recognised [5]. Research suggests that social network support impacts on a range of health and wellbeing outcomes [6], including timeliness of diagnosis [7], quality of life [8–15], experience of stress [16], anxiety/depression [13], and to impact progression [17] and survival [18]. Network member support can include sharing knowledge and experiences and facilitating access to resources [19]. Social environment, including access to and utilization of healthcare, social care and networks of support, has been highlighted as an important source of support for self-management by cancer survivors [20]. A lack of support from family and friends can lead to fatalism and sense of helplessness for people living with breast cancer [21].

The link between network support and health and wellbeing for cancer survivors is relatively well established. Drawing on Berkman and Glass [22] Kroenke [23] argues that social networks have an impact on cancer outcomes via different psychosocial pathways. These include social support, social roles, social regulation, social burden, and institutional resources [23]. For example, network support could lead to positive social relationships and interactions, lower levels of fatigue and pain interference, positive impact on self-esteem, survivor efficacy for decision making and care planning [10, 24–28]. There is some evidence that around the time of diagnosis, women with breast cancer receive significant and helpful emotional support from family and friends [29, 30]. In addition, qualitative work indicates that different network members perform different supportive functions and illustrates some of the challenges associated with mobilizing network support. A study of women with breast cancer has indicated that participants needed to make changes to the structure of their networks and how they engaged with different network members in order to cope with treatment and access support that was acceptable to them [31]. The positive impact of networks could be linked to structural network characteristics such as size and diversity of the network, and how networks mediate influence by other network members (contagion) [22, 23]. Specifically, there is evidence that larger networks can have protective effects against functional impairment, quality of life, and overall survival [10, 32, 33]. However, the relationship between the characteristics of network members, the types of support they provide, and how such support is moblised in different context is not well understood for people living with and beyond cancer.

In order to harness the potential of social networks for self-management support, there is a need to understand patterns of support, identify gaps and link such an understanding to self-management interventions [34]. Previous research has utilised qualitative and quantitative methodologies, and demonstrated the value of adopting a network approach (where network member contributions are seen as a part of a system rather than as dyadic relations) in illuminating network processes and their impact on accessing support [10, 35–37]. Networks are here conceptualised as the range of formal (e.g. healthcare professionals, social care professionals) and informal relationships (e.g. neighbours, friends, colleagues, close and distant family members, peer and community group members, partners, acquaintances) who contribute to the different types of everyday work (e.g. illness, practical, emotional) involved in the management of a long-term condition [5, 38]. Such an approach draws on the rich literature of social networks, social support, and social capital, and aims to move the emphasis away from the actions of individuals, and the role of strong ties (e.g. partners, carers) in isolation, and develop an understanding of the structure of people’s networks (the naturally developing constellations of social relationships around individuals) and the mechanisms through which network support, understood as a collective process, is mobilised in different contexts [5, 19, 23, 34]. This includes recognising the key role that weak ties (e.g. acquaintances, hobby and community groups, neighbours, colleagues), which are easily accessible and require low levels of commitment when providing support [39, 40], make an important contribution to the collective efficacy of networks, and in mobilising support that is acceptable [19, 34, 41]. This is through extending people’s access to diverse information, resources, and experiences [39, 40], extending the overall capacity of networks for illness, practical and emotional work, de-burdening strong ties (e.g. partners, family members), and improving network capacity for doing the relational work required to manage responsibilities and identities, and navigating and negotiating relationships and changes over time [19, 34, 40]. Indeed, higher support may lead to higher burden on strong ties especially for people of lower socio-economic status [42], and people maybe selective in who they engage in their networks and for what type of support [43]. Within the context of self-management support the value of weak ties is, in part, due to such ties being considered unimportant (i.e. in terms of sense of intimacy, dependence, intensity and frequency of contact, the amount and importance of the work they do) [34, 40]. Previous research has indicated that support from diverse networks that consist of a range of different relationships, including both strong and weak ties and network members with different characteristics, have a positive impact on the quality of life, self-management of people with long-term conditions and people living with cancer [10, 28, 38, 44, 45]. Drawing on this approach, the paper addresses the following questions:

- Which network members of women with breast cancer contribute to self-management support around diagnosis and what type of support do they provide?
- Which individual and network member characteristics are related to the amount and type of support received from network members of women around the time of breast cancer diagnosis?
- Do network members act as a substitute for partners in providing support and, if so, under what circumstances?

## Methods

### Ethics statement

Ethical approval was received from the North West–Preston Research Ethics Committee (reference number 16/NW/0425). Research governance approvals were obtained from individual NHS Trusts. Informed written consent was received for participation in the study.

### Design and sample characteristics

The data were collected as part of the Macmillan HORIZONS study of recovery of health and wellbeing in adults (aged 16 or over) diagnosed with cancer. HORIZONS is a prospective longitudinal cohort study of three cancer types, including women with breast cancer diagnosed under 50 years of age. A full description of the aims and methods of the study is available [20]. Recruitment took place at 110 National Health Service (NHS) hospitals from across the United Kingdom between September 2016 and March 2019. Participants were consented to the study prior to treatment, by a research nurse or member of their clinical team. Participants consented to completing study questionnaires and the collection of information from their medical records (via case report forms). Baseline questionnaires were given at consent and completed, in most cases, prior to treatment and were returned by post to the study co-ordinating centre. The analyses presented are for the pre-treatment time period, including women who had baseline data returned by the end of May 2019.

2,763 women were identified as eligible for participation in the study and of these, 2,336 (85%) were approached to participate. 1,434 (61%) of those approached gave consent. Of those who consented, 1,404 (99%) had baseline medical records data returned and 1,202 (86%) returned a baseline questionnaire. Analysis was conducted on database version dated June 2019 (V0.1-Jun-2019).

### Measures

#### Respondent level variables

Socio-demographic data, including age, gender, ethnicity, education, marital status, household structure, household tenure (owns or rents), childcare responsibilities, and income level variables (including income and social benefits), were collected by patient-reported questionnaire. The Index of Multiple Deprivation (IMD) was calculated from postcodes. In order to assess wider health issues, respondents were asked to report on presence of a list of 25 co-morbidities. Social integration and support were measured using the HEIQ (Health Education and Impact Questionnaire) social engagement subscale (five items) [46]. Ability to self-manage was measured using the Self-efficacy for Managing Chronic Disease Scale (SEMCD) [47, 48] and the HEIQ skills and technique acquisition (five items) and self-monitoring and insight (seven items) subscales [46].

#### Network member variables

Respondents were asked to name up to 20 network members considered to have played an important role in helping them to deal with their diagnosis and/or treatment [37]. For each member, they were asked to provide: gender, relationship type (open question coded into spouse/partner, close family, other family, friend, colleague, neighbour, acquaintance, group, pet, healthcare professional), frequency of contact (at least once a week, at least once a month, at least every couple of months; less often), how far away they lived (approximately in miles; recoded as co-habits/lives close by, lives further away, lives far away). Respondents were then asked to indicate the types of support each network member provided (see outcome measures below). An additional variable of presence of a proximate child (cohabiting or living close by) in the network was constructed.

#### Outcome measures: Type of support

Building on earlier work on illness management and self-management support [36, 37, 49, 50], respondents were asked to rate the contribution of each network member (no help at all, some help, a lot of help) to three types of work: illness work (information about your illness and illness management, e.g. helping you understand health information, diet, medicines), practical work (practical help with daily tasks, e.g. running your household), and emotional work (emotional support, e.g. your wellbeing, helping you feel good, comforting you when you are worried). Responses for each network member contribution were scored as ‘no help at all’ = 0, ‘some help’ = 1, ‘a lot of help’ = 2, and were summed for each type of work and network member (spouse/partner, close family, other family, friend, colleague, neighbour, acquaintance, group, pet, healthcare professional) within the network of each respondent.

### Statistical analysis

The Kruskal-Wallis test was used to compare differences between groups, given the underlying non-normal data structure. Univariate regressions were utilized to uncover the relationships between overall help received and characteristics of both the respondent and network members, with network size as a control variable. Random effect modelling (intercept) was used to assess the associations between overall help received and multiple characteristics that were identified as significant in univariable regressions for the respondent and network members respectively. When outcome variables are not normally distributed, linear regression remains a statistically sound technique in large samples. Non-normality of the errors will have some impact on the precise p-values of the tests on coefficients, but if the distribution of the data does not include large outlier, OLS still provides good approximation [51]. In our case, our data does not include large extreme values and we use regressions to point us to indicators/variables enabling us to describe patient characteristics and behaviours in details. Random effect models were conducted in R statistical software using lme package.

## Results

### Sample characteristics

Most of the participants were 41–50 years old (69.9%, n = 840), married or in a civil partnership (58.3%, n = 701), were caring for children (58.9%, n = 707), and were white (93.3%, n = 1122). While a large number of respondents were of higher socio-economic status, (72.5% (n = 871) owned their home, 63.6% (n = 765) had a university degree or professional qualifications), a substantial proportion of the sample was on lower incomes with 27.5% (n = 331) earning less than £15,599 per year, and 27.1% (n = 325) living in less affluent areas (IMD 1 and 2). Only 7.2% (n = 86) respondents lived alone (see Table 1).

**Table 1:** Sociodemographic characteristics of participants.

	N	%
Total number of respondents	1202	100%
Ethnicity*		
** White**	1122	93.3%
** Non-white**	69	5.7%
Tenure*		
*Owns*	871	72.5%
*Rents*	290	24.1%
*Other*	27	2.2%
*Marital status* *		
*Married/in civil partnership*	701	58.3%
*Single/separated/divorced/widowed*	463	38.5%
Welfare benefits*		
** Has not received benefits**	914	76.0%
** Has received benefits (e.g. unemployment/income/working tax/housing benefit)**	191	15.9%
**Age**		
** 21–30 years**	43	3.6%
** 31–40 years**	319	26.5%
** 41–50 years**	840	69.9%
Household structure*		
** Lives alone**	86	7.2%
** Lives with immediate family**	1088	90.5%
** Lives with someone else**	6	0.5%
Yearly gross salary*		
** up to £15,599**	331	27.5%
** £15,600 and up to £31,199**	371	30.9%
** £31,200 and above**	305	25.4%
** Prefer not to say**	78	6.5%
**IMD percentiles**		
** 1**	130	10.9%
** 2**	195	16.2%
** 3**	262	21.8%
** 4**	269	24.0%
** 5**	326	27.1%
Education*		
** Compulsory or lower education**	226	18.8%
** Apprentice or further education**	147	12.2%
** Higher education**	378	31.4%
** Professional or other qualification**	387	32.2%
** None of the above**	37	3.1%
Caring for Children*		
** Yes**	707	58.9%
** No**	487	40.5%

*Missing: Ethnicity, 11 (1.0%); Tenure, 14 (1.2%); Marital status, 38 (3.2%); Welfare benefits, 97 (8.1%); Household structure, 22 (1.8%); Income, 117 (9.7%); Education, 27 (2.2%); Caring for children, 8 (0.6%).

### Who are the network members of young women at the time of breast cancer diagnosis?

A total of 12,113 network members were reported. These were mainly women (n = 8,395, 69%) and people in frequent contact with the person with breast cancer (at least once a month) (n = 9,177, 76%). Most network members were friends (n = 4,933, 41%), with partners/spouses and close family members together constituting a third of network members (n = 4,206, 35%) and more distant family members just over 11% (n = 1,340). There were a small number of colleagues (n = 636, 5%), activity groups (n = 33, negligible), and healthcare professionals (n = 630, 5%) in the networks. A small proportion of respondents had a pet in their network (n = 186, 2%).

### How much and what type of work is done by different types of network members?

Network member contribution (overall mean of scores across network members for each type of work) was highest for emotional work, followed by illness work, and lowest for practical work (Table 2). In terms of contributions made by each relationship type (mean score for each relationship type for each type of work, across all networks), it was healthcare professionals who provided highest level of illness work (1.82), followed by partners (1.31), close family members (0.83), other family members (0.80) and friends (0.78). Partners provided the highest level of practical (1.78) and emotional (1.88) work. Close family members were the second highest contributors to practical work (0.95), followed by neighbours (0.89). While all network members provided high levels of emotional work, after partners (1.88), it was pets (1.77), friends (1.67) and close family (1.65) who provided the highest amounts.

**Table 2:** Illness, practical and emotional work scores by relationship type.

	Illness work*		Practical work*		Emotional work*	
	N (%)	Mean	N (%)	Mean	N (%)	Mean
Partner	873 (7.2%)	1.31	892 (7.4%)	1.78	902 (7.4%)	1.88
Close family	3021 (24.9%)	0.83	3060 (25.3%)	0.95	3137 (25.9%)	1.65
Colleagues	617 (5.1%)	0.63	608 (5.0%)	0.38	622 (5.1%)	1.46
Friends	4624 (38.2%)	0.78	4642 (38.3%)	0.53	4776 (39.4%)	1.67
Group	33 (0.3%)	0.45	33 (0.3%)	0.36	32 (0.3%)	1.31
Health professional	620 (5.1%)	1.82	581 (4.8%)	0.13	608 (5.0%)	1.23
Neighbours	77 (0.6%)	0.68	80 (0.7%)	0.89	80 (0.7%)	1.38
Other family	1243 (10.3%)	0.80	1257 (10.4%)	0.66	1301 (10.7%)	1.55
Acquaintance	62 (0.5%)	0.63	62 (0.5%)	0.32	63 (0.5%)	1.19
Pet	166 (1.4%)	0.06	166 (1.4%)	0.08	179 (1.5%)	1.77
Overall mean		0.87		0.72		1.63
Total observation	12113 (100%)		12113 (100%)		12113 (100%)	

Note: when calculate the mean, “A lot of help”, “some help” and “no help” are recoded numerically as 2, 1, 0 respectively

*Missing: Illness work, 777 (6.4%); practical work, 732 (6%), emotional work, 413 (3.4%).

### Which respondent level characteristics are associated with the amount and type of work provided by network members?

Across most measures of socio-economic status, there was a tendency for women of higher socio-economic status to receive fewer work contributions from network members (calculated as the sum of work contributions made by all network members in each network for each type of work) (Table 3). Women with a higher education qualification received less support than women with a compulsory level of education only across all domains of work (p<0.01; p<0.01; p<0.01), and women with high income received less support across all work domains compared to women of low income (p<0.01; p<0.01; p<0.01). Women living in rented accommodation and women who received benefits received more illness (p<0.01) and emotional support (p<0.05) from network members than those who owned their home and were not receiving benefits, and women in the areas of highest deprivation received more illness (p<001) and practical work (p<001) than those in more affluent areas. Being non-white was associated with more illness support than being white (p<0.01).

**Table 3:** Univariate and multivariate regressions of respondent level characteristics related to level of illness, practical and emotional support received.

	Illness work*				Practical work*				Emotional work*			
	Univariate		Multivariate		Univariate		Multivariate		Univariate		Multivariate	
	Effect size	p-value	Effect size	p-value	Effect size	p-value	Effect size	p-value	Effect size	p-value	Effect size	p-value
Age (21–30)	3.27	<0.01			3.33	<0.01			1.08	0.09		
31–40	-1.19	0.21			-0.77	0.34			-0.97	0.12		
41–50	-1.45	0.12			-1.52	0.05	-2.08	0.03	-1.18	0.04		
Education (compulsory)	2.49	<0.01			2.59	<0.01			0.59	0.11		
Apprentice	-0.23	0.71			-0.48	0.37			-0.51	0.21	-1.02	0.03
Professional Qualification	-0.53	0.36			-0.53	0.28			-0.52	0.17		
Higher	-2.35	<0.01	-1.47	0.04	-1.40	<0.01			-1.55	<0.01	-1.66	<0.01
Ethnicity (white)	1.74	<0.01			2.02	<0.01			-0.11	0.66		
Other	2.10	0.01	2.31	0.01	0.09	0.89			0.91	0.06		
Comorbidities (<3)	1.90	<0.01			2.03	<0.01			-0.03	0.91		
3 or more	-0.25	0.77			-0.13	0.86			-0.03	0.96		
Income (lowest)	2.53	<0.01			2.53	<0.01			0.45	0.14		
Medium	-0.77	0.08			-0.59	0.11			-0.71	0.01		
High	-2.31	<0.01	-1.24	0.02	-1.61	<0.01	-1.22	0.01	-0.94	<0.01		
Accommodation (own)	1.32	<0.01			1.78	<0.01			-0.21	0.43		
Rent	1.86	<0.01			0.73	0.04			0.51	0.05		
Marital status (partner)	1.52	<0.01			2.02	<0.01			-0.03	0.92		
Single/separated/divorced/widowed	0.82	0.02	1.07	0.02	-0.09	0.76			-0.09	0.69		
Welfare benefits (no)	1.45	<0.01			1.82	<0.01			-0.11	0.70		
Yes	1.92	<0.01			1.04	0.01			0.59	0.05		
Household arrangement (living alone)	1.38	0.05			1.42	0.02			-0.32	0.49		
Living with family	0.58	0.38			0.64	0.26			0.29	0.50		
Living with someone else	-2.74	0.29			0.40	0.87			-0.43	0.80		
Caring for children (yes)	1.91	<0.01			2.56	<0.01			-0.03	0.91		
No	0.05	0.89			-1.18	<0.01	-1.31	<0.01	0.00	0.99		
IMD (most deprived)	3.01	<0.01			2.74	<0.01			0.16	0.69		
Quintile 2	-0.71	0.31			-0.71	0.21			-0.52	0.23		
Quintile 3	-1.28	0.05			-1.30	0.01	-1.53	0.01	-0.01	0.97		
Quintile 4	-1.83	<0.01			-0.39	0.46			-0.11	0.70		
Quintile 5	-1.68	0.10			-1.16	0.03	-1.42	0.02	-0.30	0.45		
Self-efficacy	0.10	0.27			0.05	0.48			0.26	<0.01		
Self-monitoring (HEIQ)	0.85	0.04			0.42	0.22			1.17	<0.01		
Skills (HEIQ)	0.88	0.02			0.55	0.07			1.32	<0.01		
Social engagement (HEIQ)	1.28	<0.01	1.04	0.03	1.12	<0.01	1.13	<0.01	1.93	<0.01	1.66	<0.01
Size of network			0.71	<0.01			0.53	<0.01			1.57	< 0.01

Notes: None of the above and missing are not reported in this table

*Calculated as the sum of each type of work done by all network members within the network of each respondent.

Respondents who had higher self-management scores tended to get more support from their network members. Higher scores of self-monitoring (HEIQ) and self-management skills (HEIQ) were associated with higher levels of illness (p<0.04 and p<0.02) and emotional work (p<0.01 and p<0.01), and higher levels of self-efficacy were associated with higher levels of emotional work by network members (p<0.01). Women who were more involved with social activities (social engagement, HEIQ) received more support across all three types of work (p<0.01; p<0.01, p<0.01). Being older was associated with less practical (p<0.05) and emotional work (p<0.04), not having caring child responsibilities was associated with less practical work (p<0.01), and not having a partner with less illness work (p<0.01).

In the multivariate analysis, and controlling for size of network, measures of socio-economic status remained significant. Higher education was associated with a lower amount of illness and emotional work received (p<0.04, p<0.01), having higher income was associated with lower amount of illness and practical work (p<0.02, p<0.01), and living in deprived areas (IMD) was associated with more practical work than living in affluent areas (p<0.01, p<0.02). In the multivariate analysis, women who were more involved with social activities (social engagement, HEIQ) received more support across all three types of work (p<0.03; p<0.01, p<0.01). Being older and not caring for children were associated with less practical work (p<0.03, p<0.01), and being non-white was associated with more illness support than being white (p<0.01). We note that number of comorbidities did not show statistically significant associations with the amount of work done by network members, and self-management variables were not significant in the multivariate analysis.

### Which network member characteristics are associated with the amount and type of work provided by network members?

Most of the network member characteristics showed statistically significant associations with all three types of work on the univariate and the multivariate levels (Table 4). Higher amounts of illness, practical and emotional work were provided by network members who were women, those in frequent contact with the respondent, those who cohabited, if they were a partner or spouse, or a child who was cohabiting or living nearby. Those who lived close by provided more illness and practical support but less emotional support than network members living further away.

**Table 4:** Univariate and multivariate regressions–network member characteristics.

	Illness work*				Practical work*				Emotional work*			
	Univariate		Multivariate		Univariate		Multivariate		Univariate		Multivariate	
	Effect size	p-value	Effect size	p-value	Effect size	p-value	Effect size	p-value	Effect size	p-value	Effect size	p-value
Gender (male)	0.81	<0.01			0.92	<0.01			1.59	<0.01		
Female	0.05	0.01	0.19	<0.01	-0.30	<0.01	0.08	<0.01	0.06	<0.01	0.17	<0.01
Proximate child of woman with cancer (no)	0.86	<0.01			0.68	<0.01			1.63	<0.01		
yes	-0.30	<0.01	-0.27	<0.01	0.54	<0.01	0.01	0.65	0.02	0.41	-0.02	0.52
Contact (frequent)	0.91	<0.01			1.00	<0.01			1.69	<0.01		
Not frequent	-0.15	<0.01	-0.15	<0.01	-0.63	<0.01	-0.35	<0.01	-0.14	<0.01	-0.13	<0.01
Distance (living very close or cohabiting)	0.88	<0.01			1.32	<0.01			1.75	<0.01		
Lives nearby	-0.04	0.19	0.02	0.21	-0.58	<0.01	-0.16	<0.01	-0.13	<0.01	0.01	0.30
Further away	-0.03	0.19	0.02	0.31	-0.74	<0.01	-0.24	<0.01	-0.15	<0.01	0.04	0.01
Far away	-0.06	0.02	0.09	<0.01	-0.83	<0.01	-0.39	<0.01	-0.14	<0.01	0.11	<0.01
Relationship (partner)	1.28	<0.01			1.77	<0.01			1.87	<0.01		
Close family	-0.47	<0.01	-0.53	<0.01	-0.84	<0.01	-0.62	<0.01	-0.23	<0.01	-0.32	<0.01
Colleagues	-0.65	<0.01	-0.71	<0.01	-1.39	<0.01	-1.21	<0.01	-0.41	<0.01	-0.52	<0.01
Friends	-0.49	<0.01	-0.61	<0.01	-1.25	<0.01	-1.03	<0.01	-0.21	<0.01	-0.33	<0.01
Healthcare professionals	0.53	<0.01	0.61	<0.01	-1.65	<0.01	-1.33	<0.01	-0.62	<0.01	-0.67	<0.01
Other family	-0.51	<0.01	-0.63	<0.01	-1.13	<0.01	-0.83	<0.01	-0.35	<0.01	-0.46	<0.01
Neighbours	-0.57	<0.01	-0.67	<0.01	-0.99	<0.01	-1.05	<0.01	-0.53	<0.01	-0.66	<0.01
Groups	-0.80	<0.01	-0.83	<0.01	-1.36	<0.01	-1.39	<0.01	-0.56	<0.01	-0.71	<0.01
Acquaintances	-0.71	<0.01	-0.69	<0.01	-1.39	<0.01	-1.14	<0.01	-0.74	<0.01	-0.76	<0.01
Pets	-1.24	<0.01	-1.31	<0.01	-1.68	<0.01	-1.75	<0.01	-0.10	<0.01	-0.20	<0.01

Notes: People in Category “None of the above and missing” are not reported in this table

*Calculated as the sum of each type of work done by all network members within the network of each respondent.

### Do network member contributions differ according to the personal circumstances of the woman with breast cancer?

In order to explore the variation in network structure and type and level of the work that network members do, we divided the sample using two key indicators related to network member support: (not) having a partner and (not) having higher education. Partners are well established as key providers of support for women with breast cancer [52], and level of education has been identified as shaping access to social support for this population [53]. Specifically, lower education has been associated with psychological symptoms and distress, poor health outcomes and mental adjustment, and unmet needs [54–58]. More broadly, education level is associated with unmet, multiple or increasing needs, experiences of health burden, and psychological health among cancer survivors [59–61]. This may be due to the role of education as an important marker of socio-economic status, mediating access to resources, services, and information, as well, as through association to health and financial literacy, fatalism and individual capacity to cope [61–65]. There is also evidence that strong social support may compensate for the adverse impact of low education for people living with long-term conditions [37]. Having a partner or not, and level of education are also practical individual level characteristics that can be easily used in assessment and thus findings about people with such characteristics can directly inform practice.

We divided the sample into four groups and these are shown in Table 5 as respondents who: a) do not have higher education and do not have a partner (nHE/nP), b) do not have higher education and have a partner (nHE/P), c) have higher education and do not have a partner (HE/nP), and d) have higher education and have a partner (HE/P). For each of the four groups we report: 1) the mean work score for each network member type for each type of work; 2) the number of network members of each relationship type, and the percent that each relationship type constitutes within each of the four groups; 3) the overall mean work levels for each type of work within each of the four groups, 4) the average size of the networks for each of the four groups; and 5) the mean of the total work done for each type of work within each of the four groups (taking into consideration the average network size) (Table 5).

**Table 5:** Work done by relationship type for women with or without higher education and with or without a partner.

	No Higher Education and No Partner (nHE/nP)		No Higher Education and Partner (nHE/P)		High Education and No Partner (HE/nP)		High Education and Partner HE/P)		
Relationship	mean	%	mean	%	Mean	%	Mean	%	p-value
**Illness work**									
**Close family**	1.07	33%	0.86	28%	0.72	24%	0.64	734 (23%)	<0.01
**Colleagues**	0.70	5%	0.66	5%	0.52	7%	0.58	209 (7%)	0.41
**Friends**	1.00	47%	0.84	38%	0.69	51%	0.61	1327 (41%)	<0.01
**Groups**	0.71	1%	0.54	<1%	0.25	1%	0.22	9 (0%)	0.50
**Health professionals**	1.77	4%	1.86	5%	1.61	6%	1.82	220 (7%)	0.04
**Neighbours**	1.10	1%	0.66	1%	0.80	1%	0.27	15 (0%)	0.02
**Other family**	1.03	10%	0.88	12%	0.60	9%	0.56	330 (10%)	<0.01
**Acquaintance**	1.00	1%	0.64	<1%	0.60	1%	0.52	23 (1%)	0.42
**Pets**	0.00	0%	0.09	2%	0.00	<1%	0.04	49 (2%)	0.77
**Partner**			1.37	9%			1.17	285 (9%)	
Overall mean	1.04		0.93		0.74		0.73		<0.01
Average network size	8.79		10.38		11.25		11.00		<0.01
**Total work (mean)**	9.41		9.79		8.53		8.18		0.10
**Total work (mean) excluding partner**			8.55				7.06		0.03
**Practical work**									
**Close family**	1.08	33%	0.98	28%	0.95	24%	0.81	23%	<0.01
**Colleagues**	0.36	5%	0.41	5%	0.38	7%	0.33	6%	0.97
**Friends**	0.59	46%	0.54	38%	0.63	51%	0.47	42%	<0.01
**Groups**	0.43	1%	0.54	<1%	0.00	0%	0.22	<1%	0.51
**Health professionals**	0.28	4%	0.12	5%	0.33	6%	0.08	6%	<0.01
**Neighbours**	1.09	1%	0.93	1%	1.00	1%	0.60	<1%	0.28
**Other family**	0.78	10%	0.66	12%	0.81	9%	0.61	10%	0.03
**Acquaintance**	0.83	1%	0.18	<1%	0.75	1%	0.29	1%	0.14
**Pets**	0	0%	0.07	1%	0.46	2%	0.04	2%	<0.01
**Partner**			1.78	10%			1.78	9%	
Overall mean	0.75		0.77		0.69		0.64		<0.01
Average network size	9.02		10.45		11.24		11.03		<0.01
**Total work (mean)**	7.17		8.13		8.75		7.14		0.14
**Total work (mean) excluding partner**			6.31				5.29		0.05
**Emotional work**									
**Close family**	1.67	33%	1.69	28%	1.58	24%	1.58	23%	0.02
**Colleagues**	1.46	5%	1.50	5%	1.55	7%	1.37	6%	0.04
**Friends**	1.75	46%	1.69	38%	1.62	51%	1.61	42%	<0.01
**Groups**	1.43	1%	1.46	<1%	1.00	<1%	1.11	<1%	0.40
**Health professionals**	1.55	4%	1.24	5%	1.38	6%	1.12	7%	<0.01
**Neighbours**	1.30	1%	1.56	1%	1.22	1%	1.00	<1%	0.32
**Other family**	1.65	9%	1.57	12%	1.52	9%	1.45	10%	<0.01
**Acquaintance**	1.33	<1%	1.11	<1%	1.60	1%	1.17	1%	0.37
**Pets**	1.71	1%	1.83	2%	2.00	2%	1.59	2%	0.03
**Partner**			1.88	9%			1.89	9%	0.66
**Overall mean**	1.69		1.66		1.58		1.56		<0.01
**Average network size**	8.78		10.66		11.35		11.33		<0.01
**Total work (mean)**	14.96		17.74		17.94		17.70		<0.01
**Total work (mean) excluding partner**			15.86				15.80		0.89

The four groups differed in terms of network size, with networks of women with nHE/nP smaller than all other groups (8.78–9.02 network members), and those of women with HE/nP networks being largest (11.24–11.35 network members). Most of the support was provided by friends and close family, but there was variation in terms of how this support was distributed. Around 50% of those providing support for women with HE/nP were friends, compared to 38% for women with nHE/P. The highest proportion of close family members providing support was in networks of women with qualifications below higher education level (highest for women with nHE/nP (33%)), and it was lowest among women with HE/P (23%).

**Fig 1:**
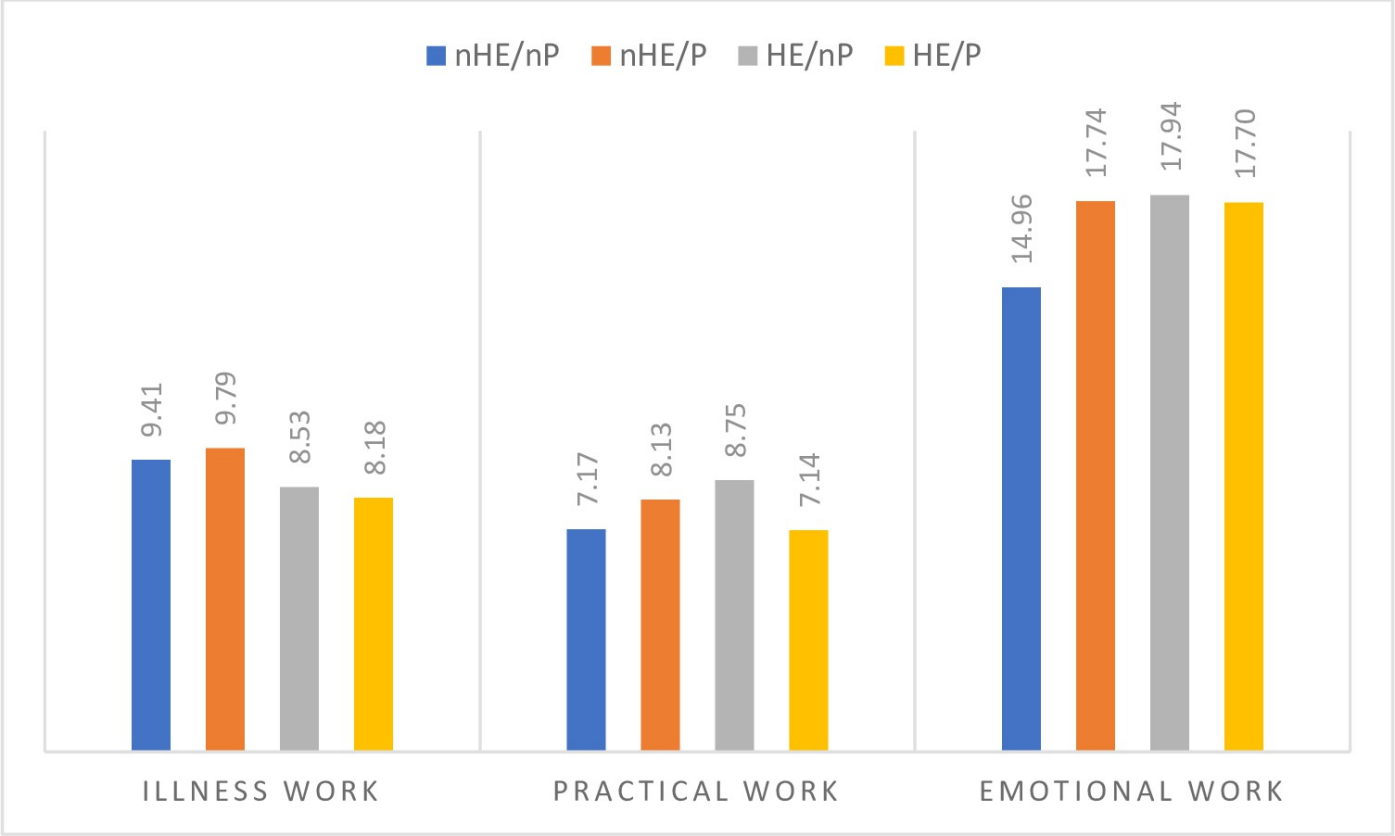
Sum of the mean work per network, for each of the four groups, taking into consideration the mean number of people from each type of relationship.

The average contribution by individual network members (overall mean) tended to be higher in networks without a partner (p<0.01; p<0.01; p<0.01). Higher contributions were made by friends (p<0.01; p<0.01; p<0.01) and other family (p<0.01; p<0.03; p<0.01), but also close family (p<0.01; p<0.01; p<0.02), neighbours, and acquaintances. Indeed, when the contributions made by partners were excluded, the total work in networks that had a partner was lower than in the networks without a partner for all types of work, except for emotional work in nHE/nP networks. The total work done by network members in HE/nP networks was highest for practical and emotional work, although for illness work it was nHE/P networks where the overall support was highest. This is visually represented in Fig 1.

There was variation in the contribution that different types of relationships made towards the three types of work. For all four groups, it was the level of network support with emotional work that was highest (14.96–17.94), followed by illness work, (8.18–9.79) and practical work (7.14–8.75). Partners made the highest level of contribution for all types of work, with the exception of illness work, where it was healthcare professionals who made the highest mean contribution (1.68–1.86). For practical work it was mainly family members (0.61–1.08) and neighbours (0.60–1.09) who made the highest (mean) contributions. All network members contributed high levels of emotional work, but it was pets (1.59–2.00) and friends (1.61–1.75) that made the highest (mean) contributions, although only a small number of respondents included pets in their network of support.

Overall, it appears that, in networks of women with higher education, the absence of support from a partner was mainly replaced by contributions of friends (both in terms of their higher average work contributions and the higher proportion of friends in such networks). In nHE/nP, partner contributions were mainly replaced through an increased number and level of contributions by close family members (with the exception of emotional work) in addition to those of friends. Thus, while close family members play a more important support role for all women without higher education (compared to those with higher education), their role is extended even further when there is no partner in such networks.

## Discussion

There has been a significant shift in health policy in the UK to patient-centred, personalised care in which an individual’s capacity and confidence to self-manage health problems are of central importance [4]. In this study we conceptualised networks as the constellations of social relationships (formal and informal) around individuals that contribute (as a collective) to the different types of everyday work involved in the management of a long-term condition [5, 23, 28]. Our research demonstrates the need to take a social network approach to self-management support, providing insight into the factors and processes that help mobilise and maintain effective network support for women with breast cancer.

Our research reveals the significant level of self-management support provided to women with breast cancer by their social network members soon after diagnosis. This confirms published research that demonstrates the important role social network members play in supporting self-management [19, 44, 66], including in the recovery of an individual’s health and wellbeing following cancer treatment [11, 67–69].

Our analysis also articulates the degree and type of work undertaken by network members of young women with breast cancer at the time of diagnosis, and the individual and network characteristics associated with self-management support. Consistent with other studies [29], network members were more involved in emotional and illness work, and less in meeting women’s practical needs. Healthcare professionals played a key role in illness work, which may reflect their input soon after diagnosis and the more structured treatment process for cancer patients compared to other long-term conditions. Partners provided practical work, with limited contribution from close family members and neighbours. Emotional work was supported by different network members, with pets and friends making the highest contributions (although there were many more friends than pets reported in people’s networks).

Previous research has reported that large, diverse networks are associated with better quality of life and illness management [10, 66]. However, networks can shrink at critical moments as people withdraw from or restrict engagement with relationships they consider inessential [70–72], with strong ties, especially family members, remaining the main source of support [72–74]. Our findings complement this by showing that friends play a key role across all networks, with their contributions similar to that of partners and close family members, while the involvement of weaker ties, such as neighbours, was limited. The emotional support that friends provide has been associated with greater wellbeing, more positive perceptions of health competence and greater self-efficacy [29]. The role of friends after diagnosis may be due to these relations being easier to negotiate and adapt than other strong ties, while protecting close family members from worry and overburden.

The presence of weaker ties within the networks indicates the acceptability of a range of support routes. Weak ties, such as hobby and community groups, and peer support groups, can still play a facilitative role in enabling wider network engagement, mobilising support that is less used, and thus alleviating burden on stronger ties [40, 75], especially during and after treatment. Previous research has demonstrated that interactions with weak ties (e.g. members of hobby and community groups, acquaintances, colleagues) make an important contribution to self-management support, health related quality of life, and well-being of people with a cancer diagnosis [38].

Peer support is defined as support offered by people who have a shared experience [76]. There is a long history of peer support within breast cancer, initiated in the 1950s in the United States, in the form of the Reach to Recovery programme and being adopted globally [77, 78]. The programme was developed to complement traditional health care services by addressing unmet psychological and practical problems following breast cancer treatment [77]. Peer support is currently offered as one-to one or group interaction, and in face-to-face, telephone or internet format [79] and around a third of women with breast cancer take up such support [80]. There is evidence that peer support can have a positive impact on negative emotions, stress management, psychological empowerment, quality of life, and health behaviours for women with breast cancer, especially where it is structured, provided one-to-one, and based in community settings, not directly related to illness management [38, 81–83]. There is also evidence that accessing peer support can be acceptable to women with cancer diagnosis where there is awareness that accessing such support is approved by medical professionals, and network members such as partners and family members [80]. Peer support can include aspects of informational, emotional and appraisal support [84], and having such peer support may also reduce the burden for these types of support from stronger ties.

Our findings further knowledge on the role of social networks in self-management support in two substantive areas. First, our study contributes towards developing a clearer understanding of the factors and mechanisms involved in mobilising network support, illuminating the normative and contextual conditions in which women with cancer and their network members negotiate the support provided [19, 75]. We found that individual and network-level characteristics shaped the amount and type of network engagement. The important contribution of partners to support is well known [31, 75, 85]. However, in our study, having a partner only translated into higher overall network support for women who did not have higher education. In networks of women with higher education, those without a partner received a higher amount of network support. This suggests that partners may play different roles in negotiating network engagement, mobilising higher levels of support from network members, especially the family, in networks of women without higher education. They may also protect other network members, increasing pressure on themselves and their partners in networks of women with higher education [75, 86].

Network members in frequent contact with women recently diagnosed with cancer, and those co-habiting or living nearby, provided a higher amount of support. Living with a cancer diagnosis can put intense pressure on these network members compared with the easier communications and relationship negotiations that occur at a distance [87]. Higher amount of relational work, (the work of negotiating relations in terms of what is acceptable and valued for the self and others), may be needed with proximate network members, which may have a negative impact on emotional well-being [88, 89]. Partners may also support the relational work needed within networks. For women with higher education, they may help sustain network support over time by protecting other network members after diagnosis. Overall, higher levels of support were received by women who were younger and had caring responsibilities for children, indicating how the understanding of need and the justification of support within networks is shaped by the wider framing of social roles, responsibilities and values.

Second, our findings reveal those most at risk of limited social network support. This is particularly the case with the substitutability of partners by other network members, (the degree to which other network members contributed more or different types of support in the absence of a partner) [5, 36]. Substitution of support may be more pertinent for younger women who are more likely to be in newer relationships/not be in a relationship and have less established and more fluid social support networks [90]. In this study, network members responded to the absence of a partner in supporting illness and practical work. However, for emotional work, only networks of women with higher education responded positively to the absence of a partner. There were also limits to the levels of substitutability available: while the level of contribution of most network members was higher in networks without a partner, the overall level of support was higher in networks of women with higher education, but lower for women without higher education (mainly due to the smaller size of such networks). This suggests that the absence of partner support was replaced by contributions from friends for women with higher education and by close family members and friends for women without higher education, and that women with higher education are able to mobilise alternative support more effectively. Women without higher education and without a partner are most at risk of lack of support, especially in terms of emotional support, with their close family members likely to be put under more intense pressure than those of the other groups.

The four groups explored in our study offer a useful heuristic through which plausible theories can be developed of how individual and network-level mechanisms for mobilising network support for women with breast cancer may interact with and co-shape the availability of support after diagnosis. Women without higher education who did not have a partner were most at risk of low support. These women were well supported in their illness-related needs, particularly by healthcare professionals. However, this support is typically available only during treatment and is unlikely to provide sufficient support for practical and emotional needs, where the level of network support for this group was lowest. These women are likely to have higher network member turnover, reduced support over time [91], and network members of lower SES with relatively limited material resources, time, and flexibility, and thus less capacity to provide support [92, 93]. Additionally, women with cancer may find it difficult to accept such support even when available due to the awareness of the pressure it is likely to put on close people (such as family and friends) who they care for. People with such characteristics may require access to additional resources that offer access to emotional and practical support.

Our findings have implications for wider policy agenda on personalised care and self-management. The imperative for delivering tailored, personalised healthcare systems, particularly for those diagnosed with cancer, is becoming well established in the UK [2–4]. Personalised care, however, needs to be co-created within the individual within the context of their social network. Relatedly, it is important to attend to the supportive role of social networks and to identify those who lack network support for self-management across the cancer pathway. Adopting a social network approach illuminates network engagement and the development of collective efficacy [34, 41, 94, 95], helping extend and complement the individual-centred aspects of self-management which focus on self-efficacy.

### Strengths and limitations of the study

The strengths of the study include the large and representative nature of the sample. However, the sample was mainly white and had more women of a higher SES. The study focused solely on young women with breast cancer. This is a group of women who have more aggressive disease and poorer outcomes than their older peers [96]. Being at an earlier life stage, this group may also have distinct psychosocial needs and social network patterns [90]. Results therefore may not be generalisable to older women or people with other cancer types, so future analysis with other patient cohorts would be valuable. Longitudinal analysis would also be helpful to ascertain changes in support over time. These are issues that further analysis of the HORIZONS dataset [20] can address. The social network approach taken here has provided a detailed view of both the characteristics of network members and the perceived quality of support provided. The analysis offers preliminary theorisations of possible trajectories of network support for women in different circumstances by exploring the plausible interactions between constellations of individual and network level processes. While the findings offer immediate implications for practice a more nuanced understanding of the processes involved in mobilising support and how they relate to different network and individual characteristics is likely to inform the development of interventions with high level of sensitivity to individual circumstances. These would need to be further tested and refined in the next stages of the study and may include exploring interactions between key predictors of network engagement, and the development of a network typology that can help to better understand the health and well-being outcomes for women with breast cancer diagnosis [66, 88–99].

### Clinical implications

The study has important implications for the health care professional (HCP) role in supporting women with breast cancer’s self-management work and engagement with network members and resources that might be available to them. A better understanding of the patterns and characteristics of network support can inform the development of interventions better tailored to the existing structure of support for women with breast cancer, and in shaping the role and types of support that needs to be provided by HCPs. Assessment by the clinical team of the self-management needs and levels of available network support at diagnosis would enable the identification of women who are at risk of lack of support and the recommendation of alternative supportive resources. In addition, support could be given to navigation of network resources and sources of support, and to negotiation of support within different network relationships, including relations outside immediate family members and close friends [19, 34]. These implications are particularly important during the COVID-19 pandemic where HCPs face higher demand and greater complexity of care requirements, which might negatively affect capacity to support women with breast cancer. Lockdowns and social distancing measures may also restrict engagement with and support from informal network members. People with such characteristics may require access to additional resources that offer access to emotional and practical support. The availability of such support is likely to be of greater need in the context of the COVID-19 pandemic.

Based on our findings we recommend that social support is included as part of a holistic assessment of needs close to diagnosis [13] and integrated into care and support plans. This is particularly important to inform personalised care and support planning after treatment and choice of follow up care, which may include personalised stratified pathways such as supported self-management.

## Conclusion

Within the context of growing recognition of the role of social networks in self-management, this study has described the structural characteristics of the network support available to young women with breast cancer at the time of diagnosis and has characterised groups that might be more at risk of lack of such support. There is need to better acknowledge and understand how networks work as a system, rather than as dyadic relations, and especially the role of social engagement and weaker ties, in mobilizing and sustaining self-management support for women with breast cancer. This study offers evidence of the value of extending the focus of self-management support to include the collective efficacy of networks, the capacity of individuals and members of their networks to mobilise support that is acceptable [34]. Exploring collective efficacy and the mechanisms of network engagement in relation to the more common focus on individual self-efficacy is helpful for informing interventions at a time of increasing emphasis on self-management across the cancer trajectory.
